# Adjunct Hyperbaric Oxygen Therapy for Refractory Infections in Patients with Hematologic Malignancies: A Single-Center Retrospective Study

**DOI:** 10.3390/cancers18132172

**Published:** 2026-07-07

**Authors:** Kunhwa Kim, Dimitrios P. Kontoyiannis, Hycienth Ahaneku, Deborah McCue, Javier Adachi, Alessandra Ferrajoli

**Affiliations:** 1 Department of Leukemia, The University of Texas MD Anderson Cancer Center, Houston, TX 77030, USA; kunhwa.kim7@gmail.com (K.K.); hycienth.ahaneku@usoncology.com (H.A.); 2 Infectious Diseases, The University of Texas MD Anderson Cancer Center, Houston, TX 77030, USA; dkontoyi@mdanderson.org (D.P.K.); jaadachi@mdanderson.org (J.A.); 3 Division of Pharmacy, The University of Texas MD Anderson Cancer Center, Houston, TX 77030, USA; dblamble@mdanderson.org

**Keywords:** hyperbaric oxygen therapy, hematological malignancies, anti-infective treatment, invasive mold disease, BK viral infection

## Abstract

**Background**: As hyperbaric oxygen therapy has been sporadically used in combination with antimicrobial treatment for refractory infections in patients with hematologic malignancies, data on its efficacy and outcomes are limited. **Methods**: We retrospectively analyzed 55 patients with hematologic malignancies treated with hyperbaric oxygen therapy over a 10-year period at a single tertiary care center and report on patients’ clinical features, infection types, treatment responses, and survival outcomes. **Results**: The most common underlying hematologic malignancy diagnosis was acute myeloid leukemia (30 patients, 55%). The most common hyperbaric oxygen therapy indications were invasive mold disease (IMD) (35 patients, 64%), BK virus-associated cystitis (17 patients, 31%), and bacterial cellulitis (3 patients, 5%). Patients underwent a median of 10 hyperbaric oxygen therapy sessions (range, 1–45). In total, 54 (98%) of the 55 patients were evaluable for response, of whom 32 (59%) patients demonstrated a response, defined as either resolution or stabilization of the infection. Among the 35 patients with IMDs, we found that 11 patients (31%) achieved a complete response, 5 (14%) had a partial response, 6 (17%) had stable disease, and 13 (37%) patients experienced progression of their infection. In contrast, among the 17 patients with BK virus-associated cystitis, 9 patients (53%) had persistent or worsening hematuria. The two evaluable patients with bacterial cellulitis responded with resolution of the infection. We reviewed the status of the hematologic malignancies at the time of hyperbaric oxygen therapy treatment and found that the 21 patients whose hematologic malignancies were in remission tended to have a higher response rate of their infection compared to patients whose hematologic malignancies were not in remission (73% vs. 46% *p* = 0.057). Despite the response of infections, 1-year mortality in these patients remained high at 76%. **Conclusions**: Based on our experience, hyperbaric oxygen therapy, in conjunction with appropriate anti-infective therapy and debridement surgery, could benefit selected patients with hematologic malignancies, especially those whose underlying disease is controlled but experience recalcitrant bacterial or fungal infections.

## Simple Summary

Hyperbaric oxygen therapy, a procedure that increases the level of oxygen circulating in the body, is sometimes used in specialized centers in addition to surgery and antimicrobial therapy to help fight infection and improve healing of infectious lesions in patients affected by hematological malignancies. Patients with hematological malignancies are at extremely high risk of infectious complications due to their compromised immune system because of the disease itself and the treatments they undergo. In this report, we present our experience with this therapeutic modality that is the largest presented so far, and share the results observed with the goal of assisting clinicians in the identification of patients best suited for this treatment.

## 1. Introduction

Hyperbaric oxygen therapy delivers 100% oxygen at high pressure and has been historically used for the treatment of carbon monoxide poisoning, burns, decompression sickness, and crush injuries in order to improve oxygen delivery to the tissue [1,2,3]. The U.S. Food and Drug Administration has cleared hyperbaric oxygen therapy devices for several indications related to infections, such as clostridial myositis and myonecrosis, sever flesh-eating bacterial infections and refractory osteomyelitis. Hyperbaric oxygen therapy has also been used anecdotally as adjunct treatment to anti-infective treatments and surgical debridement for severe or recalcitrant infections, such as necrotizing soft tissue infections, sinusitis and refractory osteomyelitis [4,5,6,7]. Theoretically, hyperbaric oxygen therapy exerts antimicrobial and immunomodulatory effects by inducing oxidative stress and the production of reactive oxygen species, thereby enhancing immune responses and promoting healing. It has been reported that hyperbaric oxygen therapy can suppress pro-inflammatory cytokines, decrease the CD4:CD8 T-cell ratio, clear neutrophils, and reduce pathogen burden locally. Hyperbaric oxygen therapy antimicrobial effects also generate less acidosis in tissues, help antifungal activities, enhance the activity of antimicrobial agents, and facilitate healing from infection [1,2].

In patients with hematologic malignancies, fungal, bacterial and viral infectious complications remain major causes of morbidity and mortality, often more difficult to manage than in the general population due to underlying treatment- and/or disease-related immunosuppression [8,9,10]. Although hyperbaric oxygen therapy has been used as an adjunct to systemic anti-infective treatments and, when indicated, debridement surgery, published data reporting clinical evidence for its benefit remains sparce [11,12,13,14].

To that end, we conducted a retrospective study using 10-year data from a single, large tertiary cancer center to characterize the patterns of use and outcomes of patients with hematologic malignancies who received adjunct hyperbaric oxygen therapy for the treatment of infections.

## 2. Patients and Methods

We retrospectively reviewed patients with hematologic malignancies who received hyperbaric oxygen therapy for infection-related indications between December 2012 and February 2022 at The University of Texas MD Anderson Cancer Center. Patients were required to have received hyperbaric oxygen therapy in adjunction to antimicrobial therapy and/or surgical debridement. This study focused on patients using hyperbaric oxygen therapy for treatment of infections, excluding those with non-infectious, non-healing chronic wound indication. Patients included in this study were identified via institutional cohort and insurance authorization data among all patients with hematological malignancies. This retrospective review was approved by the MD Anderson Cancer Center Institutional Review Board and was conducted in accordance with the Declaration of Helsinki. For invasive mold disease (IMD), response to hyperbaric oxygen therapy was assessed using EORTC criteria [15]. Demographic, clinical, and outcome data were obtained by both electronic medical records and manual charts’ review. Chi-square and Fisher’s exact tests were used for categorical analysis. Overall survival (OS) was defined from the date of the first hyperbaric oxygen therapy session to death or last follow-up. Kaplan–Meier curves were used to demonstrate OS, and the Cox proportional hazards model was used for survival analyses. The cut-off for statistical significance was at a *p*-value of 0.05. Cox’s proportional hazards model was used to study the association of demographic and clinical characteristics with survival in univariate or multivariate analyses. All analyses were conducted using STATA version 13.1 (StataCorp LLC, College Station, TX, USA).

## 3. Results

Fifty-five patients with hematologic malignancies were treated with hyperbaric oxygen therapy, and the median age of these patients was 51 years (range 18–86). Patient and disease characteristics are summarized in Table 1.

Acute myeloid leukemia (AML) (30 patients, 55%) and acute lymphoblastic leukemia (ALL) (10 patients, 18%) were the most common hematologic malignancies. In total, 34 patients (61%) had active hematologic malignancies at initiation, 47 patients (85%) had received systemic chemotherapy within 90 days before initiating hyperbaric oxygen therapy, 24 patients (44%) had a history of allogeneic stem cell transplantation (SCT), with a median time from SCT to hyperbaric oxygen therapy of 151 days (range 27–1818), and 9 patients (16%) underwent SCT within 90 days before hyperbaric oxygen therapy initiation.

Indications for hyperbaric oxygen therapy included invasive fungal sinusitis (20 patients, 36%), BK virus-associated cystitis (17 patients, 31%), cellulitis (15 patients, 27%), complicated lung consolidation of an unknown pathogen (2 patients, 4%) and fungal endophthalmitis (1 patient, 2%). Among the 38 patients who had non-BK viral infections, 10 (26%) patients were identified as having disseminated infection at the time of initiation of hyperbaric oxygen therapy, and 29 patients (76%) had uncontrolled infection, defined as progression or no improvement of disease despite 7-day antifungal treatment and >96 h of antibiotic treatment [16]. Nineteen of these patients (50%) had debridement surgery prior to initiation of hyperbaric oxygen therapy.

Thirty-five (64%) patients had IMD. Mucormycosis was the most common type of IMD (21 patients, 60%) and it was caused by Mucor species in 12 patients and Rhizopus in 9 patients (12 patients had sinusitis, 7 cellulitis and 2 pneumonia). Other types of IMD were *Fusarium* in 7 patients (20%; 3 patients had sinusitis, 3 cellulitis, 1 ophthalmitis), *Aspergillus* in 3 patients (9%; 2 patients had sinusitis, 1 cellulitis), and *Scedosporium* in 1 patient (3%; sinusitis), while suspected fungal infection without a pathogen identified accounted for the remaining cases of IMD in 3 patients (9%; 2 patients had sinusitis and 1 cellulitis).

Overall, 17 patients (31%) had BK-virus cystitis, 6 (35%) of them also had BK viremia at time of hyperbaric oxygen therapy initiation and 12 (71%) patients had received systemic therapy (consisting of intravenous cidofovir) against the BK virus before initiation of hyperbaric oxygen therapy. Moreover, 14 out of 17 patients with BK-virus cystitis had a prior history of SCT, and the median time from SCT in these patients was 77 days (range 27–222).

The three remaining patients that received hyperbaric oxygen therapy had bacterial cellulitis (all three in the toe area, and all three received intravenous antibiotics concomitantly with hyperbaric oxygen therapy).

The median number of hyperbaric oxygen therapy sessions per patient in the 55 patients studied was 10 (range 1–45), with a median treatment duration of hyperbaric oxygen therapy of 19 days (range 1–109). The duration of each session was approximately 60–90 min depending on the ability of the patient to tolerate the treatment, and hyperbaric oxygen therapy was administered via a hood or nose device in a Multiplace Chamber accredited by the Undersea Hyperbaric and Medical Society. Fifty-three patients (96%) received hyperbaric oxygen therapy in an inpatient setting, and two patients received it as outpatients. The median length of follow-up is 61.6 months (95% confidence interval (CI) 29.6–106.9 months).

Overall, 54 (98%) of the 55 patients were evaluable for response, and 32 (59%) patients demonstrated a clinical response, defined as either resolution or stabilization of the infection. Resolution of infection was observed in 15 patients (28%), and stabilization was documented in 17 patients (31%). One patient with bacterial cellulitis was not evaluable for response due the patient being lost to follow-up before sufficient information to assess response was available.

Among the 35 patients with IMDs, we reviewed each patient’s response of the infection and defined it in collaboration with infectious diseases experts according to EORTC criteria, and found that 11 patients (31%) achieved a complete response of their infection, 5 (14%) patients had a partial response, 6 (17%) patients had stable disease, and 13 (37%) patients experienced progression of their infection (Table 2).

Response of infection (complete and partial response) in patients with IMD according to the mold type was *Mucorales* 10/21 patients (48%), *Fusarium* spp. 3/7 patients (43%), *Aspergillus* spp.—3/3 patients (100%) and *Scedosporium*—0/1 patient (0%).

Response of infection (complete and partial response) in patients with IMD who received surgical debridement prior to initiation of hyperbaric oxygen therapy was 11/19 patients (58%); responses consisted of complete response of infection in 7 patients (37%) and partial response of infection in 4 patients (21%).

Response of infection (complete and partial response) in patients with single-site IMD was found in 9/19 patients (47%), where responses consisted of a complete response of infection in 5 patients (26%) and partial response of infection in 4 patients (21%).

Among the 17 patients with BK virus-associated cystitis, 2 patients (12%) achieved complete clearance of BK viremia, clinical symptoms of cystitis and hematuria, 6 patients (35%) showed an improvement in hematuria only with a declining BK viral load, and 9 patients (53%) had persistent or worsening symptoms of cystitis and hematuria. The two evaluable patients with bacterial cellulitis responded with a resolution of the infection (Table 2).

We reviewed the status of the hematologic malignancies at the time of hyperbaric oxygen therapy treatment and found that the 21 patients whose hematologic malignancies were in remission tended to have a higher response rate of their infection compared to patients whose hematological malignancy was not in remission (73% vs. 46% *p* = 0.057). Forty-seven patients (85%) were either receiving intensive chemotherapy at the time of hyperbaric oxygen therapy or underwent an SCT within 90 days of initiation of hyperbaric oxygen therapy, and we found that these two groups of patients had lower rates of infection stabilization (51% vs. 91%, *p* = 0.019) and infection resolution (21% vs. 55%, *p* = 0.054).

Among the 38 patients with non-BK virus-related infections, the diagnosis of AML as the underlying hematologic malignancy was associated with a marginally lower infection response rate of infection compared to patients with other types of hematologic malignancies (23% vs. 53%, *p* = 0.083). Similarly, patients receiving intensive chemotherapy at the time of hyperbaric oxygen therapy and/or SCT within 90 days of initiation of hyperbaric oxygen therapy exhibited a marginally lower infection response rate than those who were not receiving treatment for their hematologic malignancies (27% vs. 71%, *p* = 0.072).

**Figure 1:**
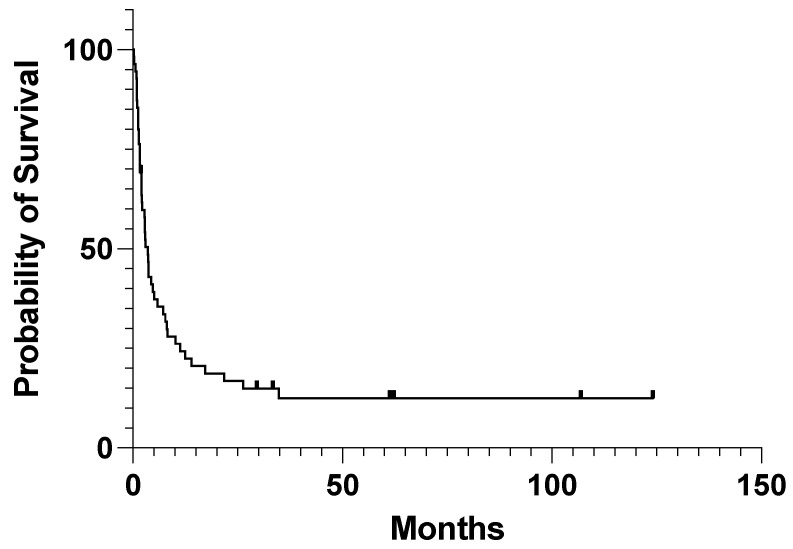
Overall survival (OS) for all patients.

Notably, although a response of infection was observed in more than half of the patients receiving hyperbaric oxygen therapy, mortality among these patients remained high. Mortally rates in the study population were 15% at 30 days, 50% at 90 days, and 76% at 1 year. At the time of hospital discharge, 64% of the patients were discharged to home, 20% of the patients were transferred to the hospice and 13% of patients died during the hospitalization. Documentation of causes of death was available for 45 patients; both hematologic malignancy-related complications and infection-related complications were the most commonly indicating causes of death, each accounting for 58% of the cases and, therefore, making it difficult to determine the individual role of each component listed. The infection for which the individual patient was receiving hyperbaric oxygen therapy treatment was indicated as directly contributing to death or to a transition in goals of care toward hospice care in five patients (11%). The median OS was 3.5 months for the overall patient cohort (Figure 1). Resolution of infection after hyperbaric oxygen therapy was associated with better survival compared to no resolution of infection (median survival in the 15 patients with resolution of infection was 14.0 months vs. 2.1 months in the patients without resolution of the infection, HR 0.28, 95% CI 0.13–0.57, *p* = 0.001). Importantly, survival was significantly associated with the underlying hematologic malignancy status, and the 21 patients whose hematologic malignancy was in remission had a better OS than those patients with an active hematologic malignancy (8.1 months vs. 2.1 months, HR 0.47, *p* = 0.017). The type of infection that was being treated with hyperbaric oxygen therapy (e.g., fungal sinusitis, BK cystitis, cellulitis) was not significantly associated with a difference in OS. There was no difference in survival associated with the various types of underlying hematologic malignancies. Similarly, neither the history of prior SCT nor the time since SCT (<1 year vs. ≥1 year or <90 days vs. ≥90 days) had a significant impact on survival. The presence of neutropenia at the time of initiation of hyperbaric oxygen therapy was not associated with a difference in survival. Importantly, hyperbaric oxygen therapy response with a resolution of infection (HR 0.28, 95% CI 0.14–0.60, *p* = 0.001) or controlled infection with any type of response (HR 0.23, 95% CI 0.11–0.48, *p* < 0.001) and the status of underlying hematologic malignancies (defined as remission or not, HR 0.42, 95% CI 0.22–0.80, *p* = 0.008) were independently associated with improved survival. Notably, patients who responded to hyperbaric oxygen therapy and were in remission from the underlying hematologic malignancies had the best outcomes, with a median OS of 7.7 months (HR 0.11, 95% CI 0.05–0.26, *p* < 0.001) (Figure 1).

## 4. Discussion

Our retrospective study presents a large single-center experience evaluating hyperbaric oxygen therapy as adjuvant treatment to antimicrobial agents and surgical debridement in patients with hematologic malignancies and severe infections treated in a tertiary cancer center setting. Consistent with previously published smaller studies, our findings support the feasibility and potential adjunctive benefit of hyperbaric oxygen therapy to antimicrobial treatment and surgical debridement in selected patients with hematological malignancies suffering from refractory infections [12,17,18,19], as we found that hyperbaric oxygen therapy when given as part of a multimodality approach was associated with a response rate of infection of 59%. Most hyperbaric oxygen therapy recipients in our series were critically ill patients being treated in the inpatient setting, with a high proportion of them undergoing chemotherapy or having recently undergone SCT; still, despite the severely immunosuppressed status, we observed that infection resolution or stabilization was achieved in a substantial proportion of patients, and that a majority of them were discharged home. Nevertheless, it is important to acknowledge that long-term survival was poor in our patients, with a median survival duration of only 3.5 months, and 30-day, 90-day and 1-year mortality rates of 15%, 50% and 76% respectively. The observed survival results highlight the high-risk nature of the population studied and the substantial cancer- and infection-related mortality.

Our results are in agreement with previously published smaller series in underscoring the critical role played by the status of the underlying disease, particularly remission versus active hematologic malignancy, in determining survival. We observed that patients whose hematologic malignancy was in remission at the time of initiation of hyperbaric oxygen therapy were having significantly longer survival, suggesting that hyperbaric oxygen therapy may be most beneficial to these patients and that patients’ hematological recovery and degree of immune suppression are important factors to be considered when selecting patients to undergo hyperbaric oxygen therapy. Although limited by the small sample size and by the heterogeneity in the type of underlying hematological malignancy, our data supports the knowledge that the immune status influences hyperbaric oxygen therapy, with a trend observed toward a lower response rate among patients with a recent history of SCT, patients receiving intensive chemotherapy at the time of hyperbaric oxygen therapy and patients with evidence of relapsed and/or refractory underlying hematological malignancies.

We acknowledge that while this study was retrospective in nature and, therefore, not designed to establish causality, the observed associations suggest that hyperbaric oxygen therapy may serve as salvage or adjunctive therapy for the treatment of infections in selected patients with hematologic malignancies, particularly those patients whose hematological malignancy is in remission or patients that are showing evidence immune recovery. However, in order to provide a balanced opinion given the high resource demands and potential toxicities of hyperbaric oxygen therapy, especially in patients who are frail, hospitalized and undergoing treatment for their hematological malignancies, it is important to remind clinicians that careful patient selection is essential. Hyperbaric oxygen therapy-related adverse effects have been reported in various types of patient populations. The most common toxicities include barotrauma, oxygen toxicity, anxiety, and visual disturbances—and are reported to occur in up to 30% of patients and rise with additional sessions. These toxicities could have a significant impact on patients’ quality of life [20]. It is important to note that the availability of hyperbaric chambers is limited to selected centers and specific geographical locations, and this may explain why reports on the use of hyperbaric oxygen therapy for the treatment of infections are rare and of small size. In our series, patients needed to be transported to a nearby facility, and coordination of care was complicated because of the need to identify an appropriate time for the therapy session, and to ascertain that the hyperbaric oxygen sessions did not conflict with scheduled antimicrobial infusion, chemotherapy infusion and other diagnostic testing. Additionally, it needs to be considered that the costs associated with the administration of a series of hyperbaric oxygen therapy sessions can be substantial and deserve appropriate consideration during the decision-making process. We performed a review of the available information and identified that Medicare estimates the cost to range from $24,000 to $50,000 annually, excluding indirect burdens such as transportation, nursing care and time dedicated by caregivers [21].

We acknowledge that there are several limitations in this study, including its retrospective uncontrolled nature, single-center tertiary care setting, and small sample size, which limit the statistical power and generalizability of the findings. Furthermore, the patient population we selected, patients with hematological malignancies, is particularly challenging to study. In our population, the presence of several concurrent infections, the frequent occurrence of neutropenia and its duration and comorbidities introduce many competing risks that complicate the attribution of survival outcomes specifically to hyperbaric oxygen therapy. Response assessments were conducted through chart review, and the retrospective nature of the analysis made the attribution of response assessment to various interventions challenging, even if the evaluating team was multidisciplinary and included a hematologist/oncologist with expertise in the management of patients with hematological malignancies, along with a clinical pharmacist with both hematology/oncology and infectious disease training and infectious disease specialists. Additionally, heterogeneity in infection types, hematologic malignancies subtypes, and supportive care interventions present within the population we studied further complicated direct comparisons across patient groups.

## 5. Conclusions

From our experience, we conclude that hyperbaric oxygen therapy can be a useful adjunct modality as part of a multimodal strategy that includes early effective antibacterial or antifungal therapy and, when indicated, early surgical debridement followed by the addition of hyperbaric oxygen therapy. Based on our results, we derive that a single site of infection and achievement of remission of the underlying hematologic malignancy are particularly important for the achievement of response of infection. It is our understanding that this is the first retrospective study investigating the effect of hyperbaric oxygen therapy with a specific focus on patients with hematologic malignancies. Although various clinical scenarios were seen among our patients, it appears that both bacterial and fungal infections could benefit from the addition of hyperbaric oxygen therapy to ongoing antibacterial and antifungal therapy, but the benefit observed in patients with BK virus cystitis was less clear.

In conclusion, in the absence of prospective studies or registries, and in view of the logistical challenges and expense associated with the administration of hyperbaric oxygen therapy, we recommend that a multidisciplinary team comprising a hematologist/oncologist, surgeon, infectious disease specialist, and (when indicated) surgeons perform careful patient selection prior to initiating a complex multimodality treatment approach that includes hyperbaric oxygen therapy.

## Figures and Tables

**Table 1:** Patients’ clinical characteristics.

	Hyperbaric Oxygen Therapy-Treated Patients (*n* = 55)
Age, median (range) years	51 (18–86)
Age ≥ 65 years, *n* (%)	14 (25)
Sex (male), *n* (%)	38 (69)
Diabetes, *n* (%)	17 (31)
Neutropenia upon hyperbaric oxygen therapy, *n* (%)	35 (64)
Diagnosis, *n* (%)	
Acute myeloid leukemia	30 (55)
Acute lymphoblastic leukemia	10 (18)
Non-Hodgkin lymphoma	4 (7)
Myelodysplastic syndrome	4 (7)
Myeloproliferative neoplasm	3 (5)
Biphenotypic leukemia	2 (4)
Chronic myeloid leukemia	2 (4)
Disease status (hematologic malignancies), *n* (%)	
Active disease	34 (62)
In remission	21 (38)
Systemic chemotherapy within 90 days of hyperbaric oxygen therapy, *n* (%)	47 (85)
Hx of SCT, *n* (%)	26 (47)
Type of SCT	
Allogeneic SCT	24/26 (92)
Autologous SCT	2/26 (8)
SCT within 1 year	17/26 (65)
SCT within 90 days	9/26 (35)
Type of infection, *n* (%)	
Invasive mold disease	35 (64)
BK-virus cystitis	17 (31)
Bacterial cellulitis	3 (5)

**Table 2:** Patient responses by infection type and hematological malignancy remission status.

**Invasive mold disease**	***n* = 35 (%)**
Complete response (EORTC)	11(31)
Partial response (EORTC)	5 (14)
Stable disease (EORTC)	6 (17)
**BK-virus cystitis**	***n* = 17 (%)**
Clearance of viremia	2 (12)
Declining viremia	6 (35)
**Bacterial cellulitis**	***n* = 2 (%)**
Resolution	2 (100)
**Hematological malignancies in remission**	***n* = 21 (%)**
Resolution or stabilization	15 (73)

## Data Availability

Data supporting this study will be made available upon request by the corresponding author.
